# Geographic variations in driving time to US mental health care, digital access to technology, and household crowdedness

**DOI:** 10.1093/haschl/qxad070

**Published:** 2023-12-01

**Authors:** Sophia N D Negaro, Rachel M Hantman, Janice C Probst, Elizabeth L Crouch, Cassie L Odahowski, Christina M Andrews, Peiyin Hung

**Affiliations:** Arnold School of Public Health, University of South Carolina, Columbia, SC 29208, United States; Department of Psychology, University of South Carolina, Columbia, SC 29208, United States; Arnold School of Public Health, University of South Carolina, Columbia, SC 29208, United States; Rural and Minority Health Research Center, Arnold School of Public Health, University of South Carolina,Columbia, SC 29210, United States; Arnold School of Public Health, University of South Carolina, Columbia, SC 29208, United States; Rural and Minority Health Research Center, Arnold School of Public Health, University of South Carolina,Columbia, SC 29210, United States; Arnold School of Public Health, University of South Carolina, Columbia, SC 29208, United States; Rural and Minority Health Research Center, Arnold School of Public Health, University of South Carolina,Columbia, SC 29210, United States; Arnold School of Public Health, University of South Carolina, Columbia, SC 29208, United States; Arnold School of Public Health, University of South Carolina, Columbia, SC 29208, United States; Rural and Minority Health Research Center, Arnold School of Public Health, University of South Carolina,Columbia, SC 29210, United States

**Keywords:** rural health, mental health, access to care, geographic disparities, travel burden, telemedicine, digital access to telecommunication tools

## Abstract

Rural residents face significant barriers in accessing mental health care, particularly as the demand for such services grows. Telemedicine has been proposed as an answer to rural gaps, but this service requires both access to appropriate technology and private space in the home to be useful. Our study documented longer travel time to mental health facilities in rural areas and greater barriers to digital devices for telemedicine access in those same areas. However, urban areas demonstrated greater household crowdedness than rural noncore areas when looking at private space within the home. Across ZIP Code Tabulation Areas located more than an estimated 30 minutes from the nearest outpatient care, 675 950 (13.1%) rural households vs 329 950 (6.4%) urban households had no broadband internet. The current Affordable Connectivity Program should target mental health–underserved communities, especially in rural America, where the scarcity of digital access compounds travel burdens to mental health care.

## Introduction

Lack of access to mental health service facilities in rural communities is a longstanding health issue affecting millions of Americans.^1^ Approximately a quarter (23.0%) of adults living in nonmetropolitan areas have been diagnosed with a mental illness.^2^ A similar prevalence is reported in urban counterparts (20.2%); however, rural residents are less likely to receive mental health treatment services from specialized professionals and encounter greater barriers to access.^1,3^ Disparity in mental health treatment access between rural and urban communities has been attributed to multiple factors, including greater stigma surrounding treatment for mental illness, shortages in mental health facilities and professionals, and various transportation barriers.^1,3^

Barriers to access in mental health treatment negatively impact individuals, families, and communities, leading to poor outcomes. In rural areas, these outcomes can include higher rates of depression and substance use, as well as increased risk of suicide.^4–7^ While treatment for these illnesses and other mental health diagnoses plays an essential role for positive long-term health outcomes,^7^ in rural communities substantially longer temporal distances to services often hinder recovery.^4^ This barrier worsens when considering other barriers to access specific to rural areas. Rural communities typically lack the necessary health care providers, with two-thirds of mental health professional shortage areas located in rural areas.^8,9^ Additionally, public transportation in rural areas is extremely limited, requiring residents to use automobiles and possess legal driver's licenses.^3^ When distances between residences and services increase, travel burden increases substantially more for those who must drive. Complicating this barrier further, residents in rural areas continue to lack insurance at higher rates when compared with urban residents.^10^ Given the compounding effect of travel burden and the increasing mental health risks and worsening outcomes, examining specific service facilities' accessibility is crucial to addressing barriers in access.

The use of telecommunication technologies to mitigate distance barriers to mental health services has increased.^11,12^ Commonly referred to as telehealth, the remote treatment strategy has various modalities that enable patients to better access treatment. Modalities include synchronous videoconferencing or telephone-delivered therapy and asynchronous mental health apps or internet-delivered programs.^13^ A recent meta-analysis comparing telehealth and in-person mental health services found similar effectiveness in reducing mental health symptoms across both treatment modalities,^14^ but 1 small rural study actually found that hybrid telemedicine increased effectiveness through better timeliness of patient care compared with only in-person services.^15^ Research also suggests that rural residents would be more interested in telehealth than their urban counterparts.^16^ This may be attributed to the increase in patient privacy that telehealth for mental health care might allow for in rural communities.^17^ Public and personal stigma surrounding the use of mental health services has been a well-documented barrier for access to services in small rural communities.^18–21^ Providing services that allows residents to engage in therapy in a private way can help mitigate the barrier.

While research demonstrates the potential benefits of digital mental health to mitigate barriers to access for rural areas, there are 2 significant barriers to its use. First, broadband access and digital device ownership remain lower in rural areas when compared with urban areas.^2,19,22^ Broadband is typically required to ensure a stable connection and to limit interruptions for videoconferencing modalities of telehealth.^20^ As of 2021, an estimated 42 million Americans did not have the ability to obtain a broadband internet connection.^21^ Second, privacy in a home or workspace to interact with digital mental health services is required to benefit from treatment.^16^ An estimated 14% of rural households have more than 1 person per bedroom, 1 measure of crowding.^23^ While there is extensive information available regarding broadband availability,^24^ little research has examined the combination of broadband availability (or its absence) and household structure (sufficient number of rooms to allow patient privacy) as barriers to services utilization.

Prior research examined geographic access to mental health facilities across the United States.^25–27^ However, studies simultaneously examining geographic access and the feasibility of digital access to mental health care are lacking. To address this gap, we conducted a national study to examine geographic access to various mental health settings—including outpatient and inpatient treatment facilities, as well as any mental health facility—and digital access to technology and internet, and household crowdedness across urban, large rural, and small or isolated rural communities.

## Data and methods

Our analysis used ecological data and was exempt from the authors' university institutional review board. We followed the Strengthening the Reporting of Observational Studies in Epidemiology (STROBE) guidelines for cross-sectional studies.^28^

### Data

ZIP Codes Tabulation Areas (ZCTAs) were the unit of observation. ZIP codes denote postal routes; ZCTAs aggregate census data within an area most closely matching each route.

The 2021 Substance Abuse and Mental Health Services Administration (SAMHSA) Behavioral Health Treatment Services Locator was used to obtain the address and types of service for mental health facilities (downloaded September 29, 2021).^29^ Facilities were mapped into the appropriate ZCTA using the United States Department of Housing and Urban Development crosswalk,^30^ as some ZCTAs contain multiple ZIP codes. The full list of facilities that offered “any” mental health services included outpatient mental health facilities, community mental health centers, hospital-based psychiatric units, psychiatric hospitals, residential treatment centers, multi-setting mental health facilities, partial hospitalization programs, and day treatment facilities. Services were further characterized by residential inpatient and outpatient service settings in the locator.

Ecological information was drawn from the 2017–2021 American Community Survey (ACS).^31^ The ACS supplied demographic information, including population, household ownership of digital technology (Table S2801: types of digital access tools), and subscription broadband (Table S2801), as well as household occupants per room (Table B25014: tenure by occupants per room). More detail is provided under “Measures” below.

To ensure accurate calculations of travel time, we excluded ZCTAs with no population and those located in Alaska and Hawaii. These 2 states have unique transportation infrastructure and travel patterns, with travel time often not being solely road-based. These exclusions yielded a sample of 31 924 ZCTAs without missing values for drive time or estimated population.

### Measures

#### Dependent variables

Our analyses had 3 end points. First, we estimated drive times from the population-weighted centroid of each ZCTA to mental health facilities. Longitude and latitude were derived from a facility's address. One-way fastest drive time, in minutes (including highways and tolls) considering the historical traffic on a typical weekday at 9 Am, was estimated using the Microsoft MapQuest application (Microsoft Corporation).^32^ A greater-than-30-minute cutoff was chosen to be in accordance with the commonly noted network adequacy travel time criteria for mental health services.^33,34^

Second, household crowdedness was measured, defined as the percentage of households in a ZCTA with greater than 1 reported current occupant per room. The ACS obtains this proportion by dividing the reported current residents by the number of separate rooms in the residential/home unit, excluding bathrooms, porches, balconies, foyers, halls, or unfinished basements. The measure was used as a proxy for privacy, as confidential interactions become more difficult under crowded conditions, which can result in constrained access to telehealth.^35^

Third, we created 2 digital access measures using ACS questions regarding digital devices and broadband. The ACS asks about computer desktops, laptops, tablets, smartphones, and other portable wireless devices, as well as whether the household has a broadband subscription.^29^ Our first access measure was the proportion of households in a ZCTA possessing no digital devices; the second was the proportion of households with no broadband subscription.

#### Independent variable

Rurality was defined using the primary Rural-Urban Commuting Area (RUCA) code, with ZCTAs classified as urban (1–3; *n* = 17 466), large rural (4–6; *n* = 4715), and small/isolated rural (7–10; *n* = 9743).^36^

#### Covariates

The ZCTA characteristics that could be associated with mental health services availability and digital access include age, sex, marital status, race, ethnicity, socioeconomic status, employment, and insurance status.^26^ Population density was calculated using land size for each ZCTA because of its potential association with driving distances.

### Analysis

Using ArcGIS Pro 2.9.1 (Esri), geographic distributions and bivariate maps of drive time to mental health facilities, household crowdedness, digital access to technology, and broadband access were developed. In the maps, drive time was used at the outcome, sorted across high-, medium-, and low-tertile categories of crowdedness, digital device ownership, and broadband subscription. Tertile values for each can be found in Table 2.

Using Stata version 17 (StataCorp), we compared ZCTA-level characteristics across rurality using 1-way analysis-of-variance (ANOVA), adjusted for multiple comparisons, and compared urban–rural differences in 3 sets of the outcomes: (1) drive times, by facility type; (2) household crowdedness; and (3) household digital access to technology and internet. Multivariable median regression models were conducted to examine the associations of ZCTA-level rurality with each outcome.

In models where drive times were the key outcomes, we conducted separate median regressions for each facility type. We compared drive times with types of mental health care services, ZCTA-level digital access and crowdedness, and between large rural, small/isolated rural ZCTAs and urban ZCTAs, with a bootstrap method for handling standard errors. All models adjusted for ZCTA-level sociodemographic characteristics, unemployment rates, uninsured rates, population density, and state fixed effects to allow within-state comparisons between rural and urban areas. Using 2-sided tests, alpha was set .05. Multicollinearity was evaluated using a variance inflation factor (VIF) index, in which we did not find any evidence of potential multicollinearity across covariates (mean VIF = 1.68).

## Results

### Demographic characteristics

Of 31 924 populated ZCTAs in the continental United States, 17 466 (55%), 4715 (15%), and 9743 (31%) were in urban, large rural, and small/isolated rural areas, respectively (Table 1). Large and small rural communities had higher percentages of households that were 200% below the federal poverty line, uninsured residents, and unemployed residents than did urban communities (Table 1). Similarly, the median household income was lower for large rural ($35 155) and small rural ($34 999) communities than for urban ones ($43 039).

**Table 1.** ZIP code tabulation area characteristics, 2019 American Community Survey, by rurality.

	Urban		Large rural		Small or isolated rural		*P*
	Mean	SD	Mean	SD	Mean	SD	
Total ZCTAs	17 466		4715		9743		
Total population	271 171 573		28 233 716		22 763 361		
Population demographic characteristics, %							
Age ≥65 y	17.6	9.4	20.1	10.2	22.8	11.9	<.001
Married	50.8	13.4	52.7	13.2	55.1	13.4	<.001
Female	50.4	5.5	49.6	7.0	49.3	7.0	<.001
Race and ethnicity, %							
Non-Hispanic White	80.2	21.1	87.7	17.7	89.2	17.8	<.001
Non-Hispanic Black	10.0	17.2	6.5	15.5	4.2	12.5	<.001
Hispanic	8.7	10.7	5.4	8.8	4.9	8.4	<.001
Non-Hispanic Asian	3.4	6.7	0.6	1.4	0.5	1.5	<.001
Non-Hispanic Indigenous/Alaska Native	0.8	4.7	1.5	6.8	2.7	11.4	<.001
Household characteristics							
Total households	5921.8	6444.4	2296.3	3580.3	900.8	5520.8	<.001
Socioeconomic characteristics							
200% below federal poverty level, %	29.7	16.7	36.0	16.0	36.5	16.4	<.001
Median household income, $	43 038	14 867	35 158	8633	34 991	9274	<.001
Uninsured, %	7.9	6.8	9.1	8.1	9.2	8.4	<.001
Unemployed, %	5.1	5.0	5.1	5.7	5.2	7.5	<.001
Household occupancy							
Households with >1 occupant per room, %	2.6	4.0	2.1	4.0	2.2	4.6	<.001
Household digital access, %							
No digital tools	8.3	8.3	12.1	10.5	13.0	10.5	<.001
No broadband subscription	14.9	11.6	21.3	13.8	22.5	14.0	<.001

Source: Data obtained from the 2021 American Community Survey 5-year estimates subject tables. *P* values were calculated using 1-way analysis of variance (ANOVA) adjusted for multiple comparisons based on Bonferroni correction. The American Community Survey classified all desktops, laptops, tablets, and smartphones, along with selected computing technologies such as smart home devices and single board computers, as digital tools.

Abbreviations: SD, standard deviation; ZCTA, ZIP Code Tabulation Area.

### Geographic availability of mental health facilities

The median drive time to “any” facility offering mental health services, including community hospitals with psychiatric beds, was 13.2 minutes for urban ZCTAs, 18.5 minutes for large rural ZCTAs, and 26.2 minutes for small or isolated rural ZCTAs. Similar differences were present for inpatient and outpatient services individually (Table 2). Unlike urban ZCTAs (29.3%), a majority of large rural and small rural ZCTAs were farther than 30 minutes from inpatient psychiatric units, with 2725(57.8%) and 8152 (83.7%) ZCTAs, respectively.

**Table 2.** Median driving time in minutes from ZIP code tabulation area centroids to mental health facilities by rurality, 2019.

		Urban (*n* = 17 466)	Large rural (*n* = 4715)	Small rural or isolated (*n* = 9743)	*P*
Driving time to the nearest mental health facility					
Any mental health facility	Median (IQR)	13.2 (7.7, 20.8)	18.5 (11.7, 26.4)	26.2 (16.9, 39.0)	<.001
	Number (%) of ZCTAs >30 min	1643 (9.4)	827 (17.5)	3970 (40.8)	<.001
Any mental health facility with outpatient services	Median (IQR)	25.9 (14.5, 48.1)	48.5 (29.7, 75.2)	64.3 (40.7, 104.1)	<.001
	Number (%) of ZCTAs >30 min	7619 (43.6)	3521 (74.7)	8427 (86.5)	<.001
Any mental health facility with inpatient services	Median (IQR)	21.4 (13.7, 32.6)	33.4 (22.3, 50.3)	51.7 (35.8, 78.5)	<.001
	Number (%) of ZCTAs >30 min	5123 (29.3)	2725 (57.8)	8152 (83.7)	<.001

Source: Authors' analysis of the 2021 SAMHSA Behavioral Health Treatment Services Locator and the 2015–2019 American Community Survey. *P* < .001 calculated from Dunn's multiple comparison tests of equality-of-populations ranks.

Abbreviations: IQR, interquartile range; SAMHSA, Substance Abuse and Mental Health Services Administration; ZCTA, ZIP Code Tabulation Area.

The geographic distribution of mental health facilities by type is illustrated in Appendix A. A total of 6440 (20.2%) ZCTAs, where an estimated 13 627 715 US residents, or 4.1% of the US population, live, require a greater than 30-minute drive to any type of mental health care facility (Appendix B). By service type, 19 567 (61.3%) and 16 000 (50.1%) of ZCTAs were more than 30 minutes of driving time to a mental health facility offering outpatient mental health services and inpatient services, respectively.

### Household crowdedness and digital access

Household crowdedness was similar between ZCTAs by drive time to mental health facilities (Appendix C). Households in ZCTAs farther from mental health facilities consistently had lower digital access. The percentage of households in ZCTAs located greater than 30 minutes from the nearest facility that lacked digital tools, such as computers, smartphones, or tablets (10.8%) was much higher than the percentage of households in ZCTAs located less than 15 minutes from facilities (6.6%). Similarly, households that were more than 30 minutes from any facility were less likely to have a broadband subscription than households less than 15 minutes from facilities (19.4% and 12.5%, respectively).

**Figure 1.**
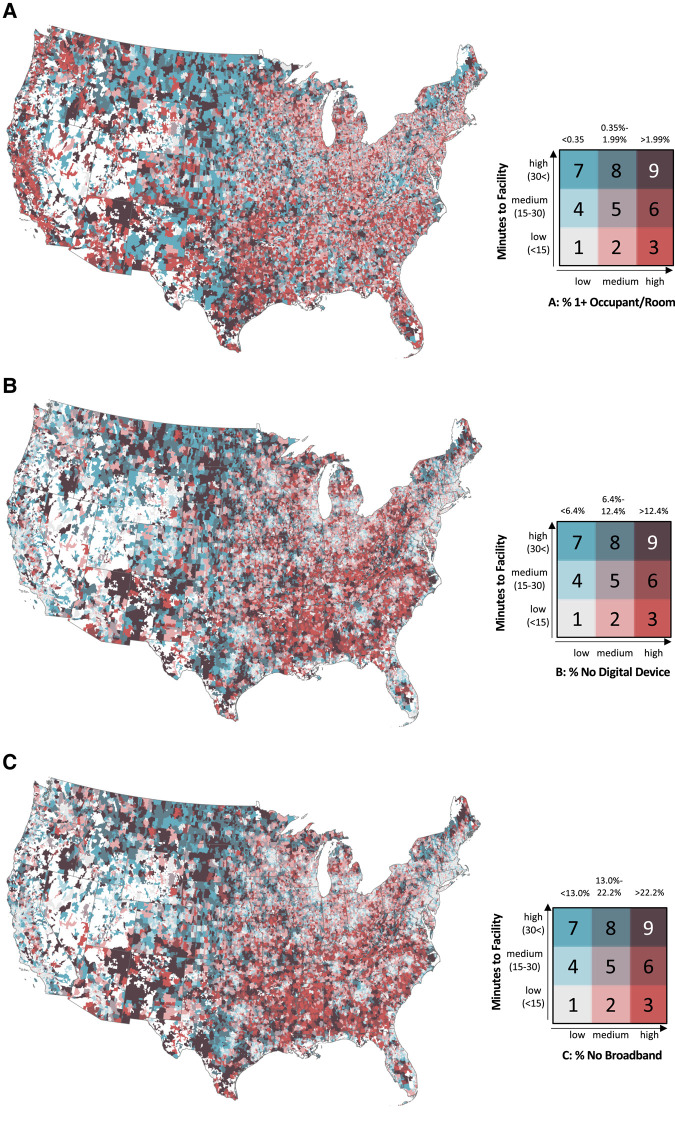
(A–C) Bivariate maps of driving times to mental health facilities and specific types of digital health access. Source: Data were extracted from the 2017–2021 ACS and the 2021 SAMHSA Behavioral Health Treatment Services Locator. Household crowdedness and unavailability of digital alternatives were calculated at the ZCTA level. Driving times from each residential ZCTA centroid to the nearest mental health service facilities were calculated using the population weighted ZCTA centroid and facility location from the SAMHSA Behavioral Health Treatment Services Locator, in minutes, using the MapQuest application. Abbreviations: ACS, American Community Survey; SAMHSA, Substance Abuse and Mental Health Services Administration; ZCTA, ZIP Code Tabulation Area.

States with the most ZCTAs where residents required a greater than 30-minute drive to any mental health facility and with the greatest household crowdedness at home included Texas (14.6%), California (8.1%), Missouri (4.3%), and Minnesota (4.0%) (Figure 1A). When looking at a greater than 30-minute drive time and digital tools, Texas (8.0%), Missouri (5.3%), West Virginia (4.9%), and Minnesota (4.2%) had the greatest percentage of ZCTAs reporting no ownership of digital tools (Figure 1B). For the proportions of statewide ZCTAs with low broadband subscriptions and longest drive times, Texas (8.9%), Missouri (5.6%), New Mexico (4.6%), and West Virginia (4.1%) were the highest (Figure 1C).

### Rural–urban disparities in drive times to mental health facilities, household crowdedness, and digital access to technology

Adjusted analyses indicated that small and large rural ZCTAs both required longer drive times to mental health care facilities and had less access to digital resources. Both large and small rural ZCTAs had longer drive times from any mental health facilities when compared with urban ZCTAs (large rural vs urban median difference: 1.7 min; 95% CI, 1.2-2.2; *P* < .001; and small rural vs urban median difference: 7.6 min; 95% CI, 7.2-8.0; *P* < .001) (Table 3). The median differences in drive times between rural and urban ZCTAs were largest when driving to facilities with outpatient services, followed by inpatient mental health services. For outpatient and inpatient mental health facilities, small rural ZCTAs had a median difference of 23.0 minutes (95% CI, 22.2-23.9; *P* < .001) and 21.4 minutes (95% CI, 20.5-22.2; *P* < .001), respectively, when compared with urban ZCTAs.

**Table 3.** Adjusted median analysis of driving time to the nearest mental health facility, household occupancy, and digital access, 2021.

	Average median difference in driving times or percentages (95% CI)		
	Urban	Large rural	Small rural
Driving time (min) to			
Any mental health facility^a^	Reference	1.68 (1.18, 2.19)^b^	7.63 (7.24, 8.01)^b,c^
Outpatient mental health facility	Reference	14.16 (12.80, 15.52)^b^	23.02 (22.15, 23.87)^b,c^
Inpatient mental health facility	Reference	7.42 (6.63, 8.20)^b^	21.38 (20.53, 22.23)^b,c^
Household occupancy			
Household crowdedness^d^	Reference	−0.04 (−0.09, 0.01)	−0.15 (−0.20, −0.08)^b^
Household digital access			
No digital tools	Reference	1.40 (1.22,1.57)^b^	2.18 (1.98, 2.37)^b,c^
No broadband subscription	Reference	2.17 (1.96, 2.38)^b^	3.02 (2.81, 3.22)^b,c^

Source: Authors' analysis of data from 2021 American Community Survey 5-year estimates subject tables and 2021 SAMSHA Behavioral Health Treatment Services Locator. *n* = 31 924 ZCTAs. Models also adjusted for proportion of residents aged 65 or older, by race, ethnicity, marital status, socioeconomic status, unemployment, insurance status, and population density.

Abbreviations: SAMHSA, Substance Abuse and Mental Health Services Administration; ZCTA, ZIP Code Tabulation Area.

^a^Any mental health facility includes outpatient mental health facilities, community mental health centers, hospital-based psychiatric units, psychiatric hospitals, residential treatment centers, multi-setting mental health facilities, partial hospitalization programs, and day treatment facilities.

^b^*P* < .001 for comparisons with urban ZCTAs, adjusted for multiple comparisons using Bonferroni correction.

^c^*P* < .001 for comparisons with large rural ZCTAs, adjusted for multiple comparisons using Bonferroni correction.

^d^Household crowdedness is measured as % ZCTA-level households with more than 1 reported occupant per living and sleeping rooms, ranging from 0% to 100%, with higher values signifying less personal space.

In addition to disproportionate travel burdens, proportionately fewer residents in both large and small rural ZCTAs had access to digital tools and broadband subscriptions when compared with urban ZCTAs. For example, households in small rural ZCTAs were more likely to lack digital tools (average median difference in proportion of households: 2.2; 95% CI, 2.0-2.8; *P* < .001) and broadband subscriptions (3.0; 95% CI, 2.8-3.2; *P* < .001) than urban ZCTAs after adjusting for ZCTA-level sociodemographic characteristics, population density, and state differences. On the other hand, despite higher rates of ownership of digital tools and broadband subscription, households in urban communities had lower household privacy than those in rural communities.

## Discussion

We found longer drive times for small and large rural communities than for urban communities across all studied mental health care facility types (any, outpatient, and inpatient services), and lower digital access via digital devices and broadband subscriptions in rural communities. In 2021, residents in rural communities had to drive an average of 26.2 minutes to any mental health care facility, 64.3 minutes to an outpatient facility, and 51.7 minutes to an inpatient facility. In addition to these increased drive times, residents of rural areas were less likely to have digital tools and broadband subscriptions, which theoretically could facilitate the substitution of virtual services for in-person mental health care. Urban households, on the other hand, had greater household crowdedness when compared with small rural areas, suggesting different challenges in telehealth uptake for mental health care between rural and urban residents.^37–39^

Our findings regarding distance to care align with previous research demonstrating poor geographic availability to mental health facilities and service providers,^26^ reflecting the lack of mental health providers in rural areas.^1,27,40,41^ This study provides a national-level examination of both travel proximity and facilitators of virtual access to mental health services, and how they vary across rurality. Our findings suggest that digital access resources are less likely to be located where they are needed most: in rural America. Yet, urban communities faced different challenges in telehealth use, given the higher household crowdedness in urban households than in small rural households.

The promise of telehealth to improve access to mental health treatment has not closed the gap in mental health care use between rural and urban populations.^42^ It is possible that inadequate access to digital tools, broadband, and privacy has been part of the reason that telehealth has not met its promise. Our research provides additional geographic clarity to the places, and thus the people, who have difficulty accessing mental health care in either site-based or virtual form. States like Texas, California, Missouri, and Minnesota, where drive times and household crowdedness are particularly high, could benefit most from tailored initiatives in rural vs urban communities by targeting the most underserved communities with different focuses. Our findings indicate the need to address privacy-related issues in more urban communities, where residents might be concerned about privacy in virtual mental health care more than in rural communities where digital access and distance to care are more of the issues. However, household crowdedness should be viewed contextually before it is viewed as a barrier. For instance, in homes with elderly or other populations who may experience technology challenges, household crowdedness may serve as a benefit, as these populations may require assistance from household members in order to facilitate telehealth use.^38,43^ Conversely, in homes where people do not need technological assistance, crowdedness may instead act as a barrier via its link to breached privacy.^38,39^

Our findings that rural areas have longer drive times and lower digital access via digital devices and broadband subscriptions, are particularly salient given recent policy changes to telehealth utilization catalyzed by COVID-19. At the federal level, the US Department of Health and Human Services authorized the extension of Medicare coverage for telehealth that was first introduced via the Coronavirus Aid, Relief, and Economic Security (CARES) Act in 2020 and set to expire in December 2024.^39^ To facilitate the use of needed mental health services in a rapidly changing health care environment, state-level policies like payment parity for telehealth services and Medicaid reimbursement for audio-only telehealth services, were adopted within the United States. Specifically for mental health care, compacts like the Psychology Interjurisdictional Compact (PSYPACT) and Medical Licensure Compact (IMLC) allow participating states to enable psychiatrists to practice across state lines via telehealth, improving the shortages of mental health professionals in states where rural and frontier area residents had to travel substantially further for mental health care.^44,45^ In fact, rural areas appeared to benefit most from these policy adoptions. In a 2023 study looking at whether policy adoption increased the odds of mental health treatment facilities offering telehealth services, rural counties were found to have significantly greater odds of having a treatment facility that offered telehealth services compared with urban facilities.^37^ However, when taking into account our findings that rural counties are less likely to have broadband connections or digital devices, increasing telehealth services without increasing accessibility for patients may be insufficient to reach individuals in need of services. This suggests that, although telehealth services are being offered more regularly across mental health providers, there is still a need to address amenity issues specific to patient access.^46^

While insurance reimbursement and coverage for telehealth could help mitigate travel burdens, it might not suffice in improving access to mental health care in rural and urban underserved communities. The uptake in telehealth has faced many challenges. One most important to our study is the cultural divide between providers and patients. Complications with urban health care professionals providing remote care to rural residents come with complexities that will need further investigation. As more than 20% of rural residents identify as American Native or people of color, delivering care with cultural humility specific to rural areas will be a needed focus for clinicians who hope to appropriately deliver high-quality mental health care.^47^

Public policy has tried to address some technical aspects of the digital divide. In 2019, the Federal Communications Commission (FCC) proposed the Rural Digital Opportunity Fund (RDOF) to improve broadband access in areas that were lacking appropriate connectivity.^48^ At that time, $20.4 billion was allocated over 10 years to “bridge the digital divide” via a 2-phase, competitive reverse auction for broadband providers. However, there were questions regarding the degree to which this provider-focused effort addressed the needs of rural communities and were being misallocated.^49^ Despite the intended application of funding to increase broadband access, our findings, that rural areas have fewer broadband subscriptions, suggest that the misallocation of funding may be a cause of continued lack of broadband access in rural areas. However, more recent policy changes may be increasing access that we were unable to measure in the dataset we used. To address the gap between the availability of broadband and actual household use of broadband, the Affordable Connectivity Program was included in the 2021 Infrastructure Act. The Act provides subsidies to help low-income families with the cost of broadband connectivity.^50^ Funds were specifically allocated for rural communities within the most recent cycles of this overall Act.^51^

Our study has several limitations, the first of which was the use of ecological data at the ZCTA level. Second, the use of self-report survey data can cause recall or response bias. Third, the cross-sectional study design also hinders our understanding of temporal trends in travel burdens to mental health care and digital access to technology after the data year. Fourth, our analyses also did not consider primary care settings as mental health facilities, although many primary care practitioners offer mental health care. Fifth, it is important to specify that, while we used drive time, this measure does not always adequately reflect travel time. Traffic, speed, detours, and other uncontrollable variations may affect the overall drive time. Sixth, household crowdedness was used as a proxy for privacy, measured by the number of households with greater than 1 reported current occupant per room. Privacy is an important element of seeking and engaging in mental health care, especially in rural areas where stigma is high. However, we did not directly measure household privacy. Finally, we did not examine physical access to broadband, but rather, whether the household subscribed to broadband services.

Continued surveillance will be needed to assess rural access to and use of telehealth services for mental health care, as well as policy-level changes that impact the use of these services. Telehealth as an adjunct to in-person care requires technological capacity and willingness to engage digitally at both the patient and the provider level. In addition, neglecting accessibility barriers for rural and low-income communities might exacerbate the mental health inequity, further disadvantaging historically underserved populations.

## Supplementary Material

qxad070_Supplementary_Data

## References

[1] Larson EH, Patterson DG, Garberson LA, Andrilla HA. Supply and Distribution of the Behavioral Health Workforce in Rural America. WWAMI Rural Health Research Center, University of Washington; 2016.

[2] Substance Abuse and Mental HealthServices Administration. *2022 National Survey on Drug Use and Health (NSDUH) releases [Internet]*. SAMHSA. Accessed October 23, 2023. Available from: https://www.samhsa.gov/data/

[3] Mattson J, Mistry D. Rural Transit Fact Book 2022. North Dakota State University; 2022.

[4] Wong H, Moore K, Angstman KB, Garrison GM. Impact of rural address and distance from clinic on depression outcomes within a primary care medical home practice. BMC Fam Pract. 2019;20(1):123. 31488051 10.1186/s12875-019-1015-7 PMC6727576

[5] Probst JC, Laditka SB, Moore CG, Harun N, Powell MP, Baxley EG. Rural-urban differences in depression prevalence: implications for family medicine. Fam Med. 2006;38(9):653–660. 17009190

[6] Musgrove R, Jackson MS, Belanger K, et al Understanding the Impact of Suicide in Rural America. National Advisory Committee on Rural Health and Human Services; 2017.

[7] Coombs NC, Meriwether WE, Caringi J, Newcomer SR. Barriers to healthcare access among U.S. adults with mental health challenges: a population-based study. SSM Popul Health. 2021;15:100847. 34179332 10.1016/j.ssmph.2021.100847 PMC8214217

[8] Rural Mental Health. Rural Health Information Hub. October 20, 2021. Accessed August 30, 2023. https://www.ruralhealthinfo.org/topics/mental-health

[9] Newton H, Beetham T, Busch SH. Association of access to crisis intervention teams with county sociodemographic characteristics and state Medicaid policies and its implications for a new mental health crisis lifeline. JAMA Netw Open. 2022;5(7):e2224803. 35838666 10.1001/jamanetworkopen.2022.24803 PMC9287760

[10] Day JC. Rates of uninsured fall in rural counties, remain higher than urban counties. United States Census Bureau. April 9, 2019. Accessed August 31, 2023. https://www.census.gov/library/stories/2019/04/health-insurance-rural-america.html

[11] Skorburg JA, Yam J. Is there an app for that? Ethical issues in the digital mental health response to COVID-19. AJOB Neurosci. 2022;13(3):177–190. 33989127 10.1080/21507740.2021.1918284

[12] Alvarado HA. Telemedicine Services in Substance Use and Mental Health Treatment Facilities (The CBHSQ Spotlight). Substance Abuse and Mental Health Services Administration; 2021.

[13] Nelson EL, Bui TN, Velasquez SE. Telepsychology: research and practice overview. Child Adolesc Psychiatr Clin N Am. 2011;20(1):67–79. 21092913 10.1016/j.chc.2010.08.005

[14] Batastini AB, Paprzycki P, Jones ACT, MacLean N. Are videoconferenced mental and behavioral health services just as good as in-person? A meta-analysis of a fast-growing practice. Clin Psychol Rev. 2021;83:101944. 33227560 10.1016/j.cpr.2020.101944

[15] Hughes MC, Gorman JM, Ren Y, Khalid S, Clayton C. Increasing access to rural mental health care using hybrid care that includes telepsychiatry. J Rural Ment Health. 2019;43(1):30–37.

[16] Weinzimmer LG, Dalstrom MD, Klein CJ, Foulger R, de Ramirez SS. The relationship between access to mental health counseling and interest in rural telehealth. J Rural Ment Health. 2021;45(3):219–228.

[17] Substance Abuse and Mental Health Services Administration. Rural behavioral health: telehealth challenges and opportunities. In Brief. 2016;9(2):1–13.

[18] Simmons LA, Yang NY, Wu Q, Bush HM, Crofford LJ. Public and personal depression stigma in a rural American female sample. Arch Psychiatr Nurs. 2015;29(6):407–412. 26577555 10.1016/j.apnu.2015.06.015

[19] Curtis ME, Clingan SE, Guo H, Zhu Y, Mooney LJ, Hser Y. Disparities in digital access among American rural and urban households and implications for telemedicine-based services. J Rural Health. 2022;38(3):512–518. 34355427 10.1111/jrh.12614 PMC9827725

[20] Substance Abuse and Mental Health Services Administration (SAMHSA). Telehealth for the Treatment of Serious Mental Illness and Substance Use Disorders. SAMHSA; 2021. Accessed December 13, 2023. Available from: https://store.samhsa.gov/sites/default/files/pep21-06-02-001.pdf

[21] Busby J, Tanberk J, Cooper T. BroadbandNow estimates availability for all 50 States: Confirms that more than 42 million Americans do not have access to broadband [Internet]. Accessed September 23, 2023. Available from: https://broadbandnow.com/research/fcc-broadband-overreporting-by-state

[22] Mehrotra A, Huskamp HA, Souza J, et al Rapid growth in mental health telemedicine use among rural Medicare beneficiaries, wide variation across states. Health Aff. 2017;36(5):909–917. 10.1377/hlthaff.2016.1461 28461359

[23] Swendener A, Rydberg K, Tuttle M, Yam H, Henning-Smith C. Crowded Housing and Housing Cost Burden by Disability, Race, Ethnicity, and Rural-Urban Location. UMN Rural Health Research Center Policy Brief; 2023. Accessed September 24, 2023. Available from: https://rhrc.umn.edu/publication/crowded-housing-and-housing-cost-burden-by-disability-race-ethnicity-and-rural-urban-location/

[24] Federal Communications Commission. Mapping broadband health in America 2023. June 2023. Accessed November 8, 2022. https://www.fcc.gov/reports-research/maps/connect2health/#ll=-67.474922,-62.226563&z=2&t=broadband&bbm=fixed_access&dmf=none&zlt=county

[25] Cummings JR, Wen H, Ko M, Druss BG. Geography and the Medicaid mental health care infrastructure: implications for health care reform. JAMA Psychiatry. 2013;70(10):1084–1090. 23965816 10.1001/jamapsychiatry.2013.377 PMC4048197

[26] Cummings JR, Allen L, Clennon J, Ji X, Druss BG. Geographic access to specialty mental health care across high-and low-income US communities. JAMA Psychiatry. 2017;74(5):476–484. 28384733 10.1001/jamapsychiatry.2017.0303 PMC5693377

[27] Ellis AR, Konrad TR, Thomas KC, Morrissey JP. County-level estimates of mental health professional supply in the United States. Psychiatric Services. 2009;60(10):1315–1322. 19797370 10.1176/ps.2009.60.10.1315

[28] von Elm E, Altman DG, Egger M, et al The Strengthening the Reporting of Observational Studies in Epidemiology (STROBE) statement: guidelines for reporting observational studies. J Clin Epidemiol. 2008;61(4):344–349. 18313558 10.1016/j.jclinepi.2007.11.008

[29] Substance Abuse and Mental Health Services Administration (SAMHSA). Behavioral Health Treatment Services Locator. Published 2021. Accessed September 8, 2021. https://findtreatment.gov/locator

[30] Office of Policy Development and Research. ZIP CODE to ZCTA Crosswalk. Accessed September 28, 2022. https://www.huduser.gov/portal/datasets/usps_crosswalk.html

[31] US Census Bureau. American Community Survey 5-year estimates. American FactFinder. Published 2021. Accessed July 8, 2022. https://data.census.gov/table? q=computer&tid=ACSST5Y2020.S2801

[32] Microsoft Corporation. MapPoint North America [Computer software]. Microsoft Corporation; 2013.

[33] Minnesota Department of Health. Provider Network Adequacy Detailed Submission Instructions Plan Year 2023 [Internet]. Minnesota Department of Health Managed Care Systems; Accessed September 23, 2023. Available from: https://www.health.state.mn.us/facilities/insurance/managedcare/docs/netadequacyinst.pdf

[34] Zhu JM, Breslau J, McConnell KJ. Medicaid managed care network adequacy standards for mental health care access. JAMA Health Forum. 2021;2(5):e210280. 34124711 10.1001/jamahealthforum.2021.0280 PMC8195259

[35] US Department of Health and Human Services. Health equity in telehealth. August 15, 2023. Accessed September 29, 2023. https://telehealth.hhs.gov/providers/health-equity-in-telehealth

[36] US Department of Agriculture. Rural-urban commuting area (RUCA) codes: documentation [Internet]. Economic Research Service. Accessed October 20, 2023. Available from: https://www.ers.usda.gov/data-products/rural-urban-commuting-areacodes/documentation/

[37] McBain RK, Schuler MS, Qureshi N, et al Expansion of telehealth availability for mental health care after state-level policy changes from 2019 to 2022. JAMA Netw Open. 2023;6(6):e2318045. 37310741 10.1001/jamanetworkopen.2023.18045 PMC10265313

[38] Svistova J, Harris C, Fogarty B, Kulp C, Lee A. Use of telehealth amid the COVID-19 pandemic: experiences of mental health providers serving rural youth and elderly in Pennsylvania. Adm Policy Ment Health. 2022;49(4):530–538. 10.1007/s10488-021-01181-z 34846613 PMC8630285

[39] US Department of Health and Human Services. Telehealth policy changes after the COVID-19 public health emergency. Accessed September 29, 2023. Telehealth.HHS.gov.

[40] Andrilla CHA, Patterson DG, Garberson LA, Coulthard C, Larson EH. Geographic variation in the supply of selected behavioral health providers. Am J Prev Med. 2018;54(6):S199–S207. 29779543 10.1016/j.amepre.2018.01.004

[41] Thomas D, MacDowell M, Glasser M. Rural mental health workforce needs assessment: a national survey. Rural Remote Health. 2012;12(4):2176. 23088609

[42] Patel SY, Huskamp HA, Busch AB, Mehrotra A. Telemental health and US rural–urban differences in specialty mental health use, 2010–2017. Am J Public Health. 2020;110(9):1308–1314. 10.2105/AJPH.2020.305657 32673109 PMC7427215

[43] Brenes GA, Danhauer SC, Lyles MF, Hogan PE, Miller ME. Barriers to mental health treatment in rural older adults. Am J Geriatr Psychiatry. 2015;23(11):1172–1178. 10.1016/j.jagp.2015.06.002 26245880 PMC4663185

[44] PSYPACTMap—Psychology Interjurisdictional Compact (PSYPACT). Accessed October 29, 2023. https://psypact.org/mpage/psypactmap

[45] Physician License | Interstate Medical Licensure Compact. Accessed October 29, 2023. https://www.imlcc.org/

[46] Clare CA. Telehealth and the digital divide as a social determinant of health during the COVID-19 pandemic. Netw Model Anal Health Inform Bioinform. 2021;10(1):26. 33842187 10.1007/s13721-021-00300-y PMC8019343

[47] Kozhimannil KB, Henning-Smith C. Racism and health in rural America. J Health Care Poor Underserved. 2018;29(1):35–43. 29503285 10.1353/hpu.2018.0004

[48] Federal Communications Commission [Internet]. FCC. Press release, Connect America Fund Phase II Auction; 2023 Feb 10 Accessed September 29, 2023. Available from: https://www.fcc.gov/auction/903

[49] Veigle A. FCC Makes Available Over $311 Million For Broadband in 36 States, While Taking Steps to Clean Up The Rural Digital Opportunity Fund Program. Federal Communications Commission (FCC); June 26, 2021. Accessed August 30, 2023. Available from: https://docs.fcc.gov/public/attachments/DOC-374406A1.pdf

[50] Federal Communications Commission. Affordable Connectivity Program. FCC; 2023.

[51] US Department of Agriculture. Biden-Harris Administration Announces Over $700 Million to Connect People in Rural Areas to High-Speed Internet. Press Release No 0126.23. June 12, 2023. Accessed September 29, 2023. https://www.usda.gov/media/press-releases/2023/06/12/biden-harris-administration-announces-over-700-million-connect

