# Supplementary material for: Geographic variations in driving time to US mental health care, digital access to technology, and household crowdedness
Source: Health Aff Sch. 2023 Dec 1;1(6):qxad070. doi: 10.1093/haschl/qxad070 (PMC10986236; doi:10.1093/haschl/qxad070)
Supplement: qxad070_Supplementary_Data [file qxad070_Supplementary_Data.zip › Supplemental Material 092023.docx]

**Supplemental Appendix**

for

Geographic Variations in Driving Time to U.S. Mental Healthcare, Digital Access to Technology, & Household Crowdedness

Appendix A: Geographic Distribution of Mental Health Facilities Overall, by Care Setting

Appendix B: Proportion of residents with Digital Access facilitators, by Driving Distance to Mental Health Facilities, 2021

Appendix C: ZIP-code Tabulation Area (ZCTA)-level Demographic Characteristics, Housing Crowdedness, and Digital Access in 2019

Appendix A. Geographic Distribution of Mental Health Facilities Overall, by Care Setting
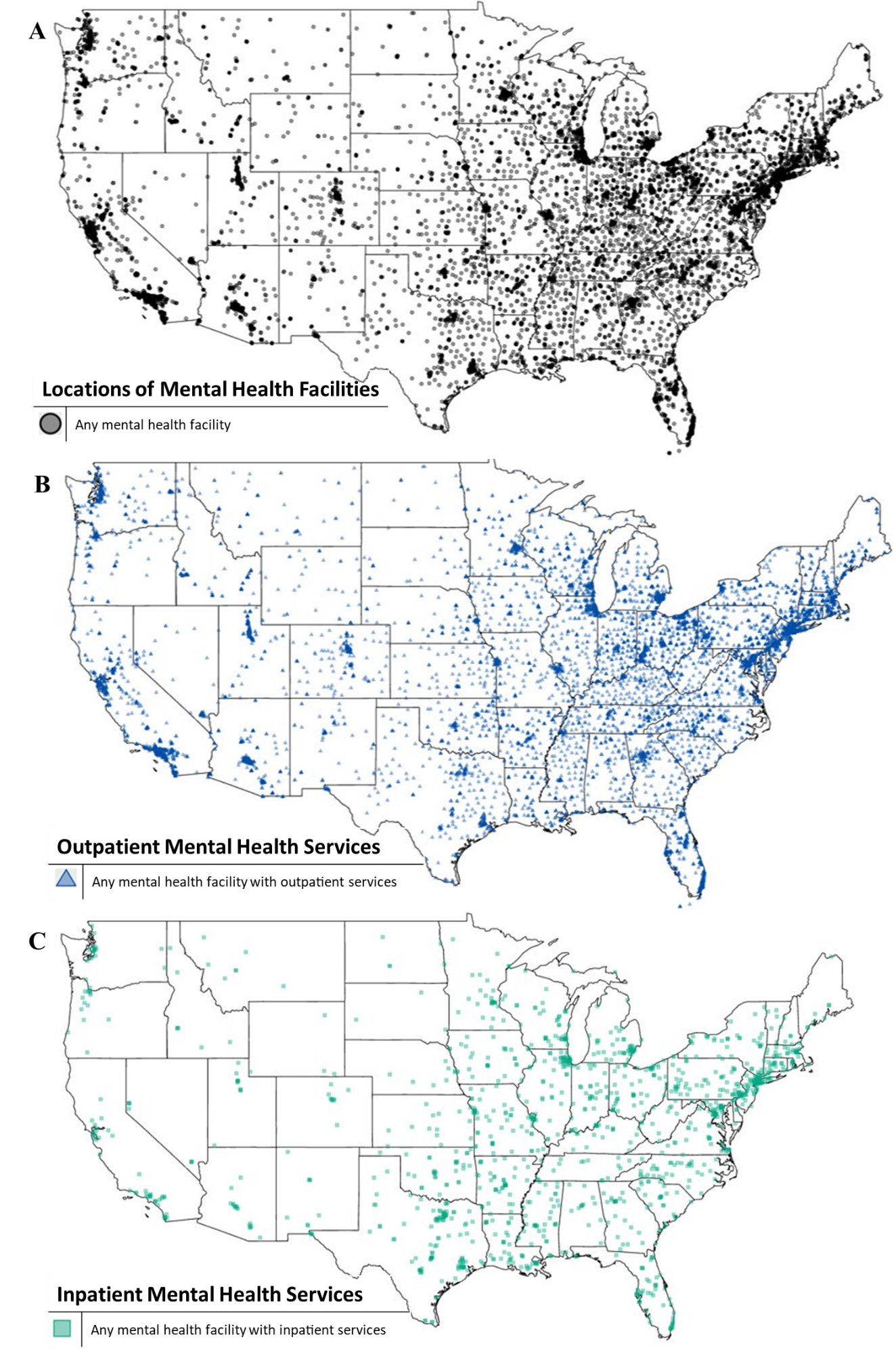
 Source/Notes: Authors’ Analysis on the data extracted from the 2021 SAMHSA Behavioral Health Treatment Services Locator. The markers represent the address for each type of facility (downloaded September 29, 2021).

Appendix B. Proportion of residents with Digital Access facilitators, by Driving Time to Mental Health Facilities, 2021

| Driving distances to the nearest mental health facility: | | | | | | | | | |
| --- | --- | --- | --- | --- | --- | --- | --- | --- | --- |
|  | <15 Minutes | | | 15-30 Minutes | | | >30 Minutes | | |
| Population (Households) | 244,916,927 (93,932,863) | | | 63,624,008 (23,919,278) | | | 13,627,715 (5,176,491) | | |
|  | Total Households (%) | Total Households in Urban (%) | Total Households in Rural (%) | Total Households (%) | Total Households in Urban. (%) | Total Households in Rural (%) | Total Households (%) | Total Households in Urban (%) | Total Households in Rural (%) |
| **Households with more than 1 occupant per room** | 3,372,158 (3.6) | 3,140,875 (3.3) | 231,283  (0.2) | 550,870  (2.3) | 412,666  (1.7) | 138,204  (0.6) | 146,771  (2.8) | 59,365  (1.1) | 87,406  (1.7) |
| **No Digital access equipment** | 6,255,976  (6.6) | 5,138,238 (5.5) | 1,117,738  (1.2) | 1,743,073 (7.3) | 1,066,302 (4.5) | 676,771  (2.8) | 561,216 (10.8) | 176,572 (3.4) | 384,644  (7.4) |
| **No broadband subscription** | 11,761,388 (12.5) | 9,814,491 (10.4) | 10,526,489  (11.2) | 3,244,804 (13.6) | 2,065,051 (8.6) | 1,179,753  (4.9) | 1,005,363  (19.4) | 329,413  (6.4) | 675,950  (13.1) |

Appendix C. ZIP-code Tabulation Area (ZCTA)-level Demographic Characteristics, Housing Crowdedness, and Digital Access

| **Total Population ^a^** | 322,168,650 | |
| --- | --- | --- |
| **Total Number of Households** | 138,485,193 | |
|  | Mean | SD |
| **Population Demographic Characteristics ^b^** |  |  |
| % Age >65 years | 19.6 | 10.8 |
| % Married | 52.4 | 13.6 |
| % Female | 49.9 | 6.3 |
| Race and ethnicity **^c^** |  |  |
| % Non-Hispanic White | 84.1 | 20.2 |
| % Non-Hispanic Black | 7.7 | 15.9 |
| % Hispanic | 7.1 | 10.0 |
| % Non-Hispanic Asians | 2.1 | 5.2 |
| % Non-Hispanic Indigenous / Alaska Native | 1.5 | 7.7 |
| **Household Characteristics ^d^** |  |  |
| Socioeconomic Characteristics |  |  |
| % 200% below Federal Poverty Line | 32.7 | 16.9 |
| Median household income | $39,509 | $13,268 |
| % Uninsured | 8.5 | 7.6 |
| % Unemployed | 5.1 | 6.0 |
| Household Occupancy **^e^** |  |  |
| % Households with more than 1 occupant per room | 2.4 | 4.2 |
| Household digital access **^e^** |  |  |
| Equipment for digital access |  |  |
| % No digital devices | 10.3 | 9.6 |
| Broadband use |  |  |
| % with digital devices but no broadband subscription | 18.2 | 13.2 |

Notes: a. Age data were derived from the 2019 American Community Survey 5-Year Estimates, Table S0101. b. Marital status and sex data were derived from 2019 American Community Survey 5-Year Estimates, Table S1201. c. Race and ethnicity data were derived from 2019 American Community Survey 5-Year Estimates, Table B03002. d. Household characteristics were derived from 2021 American Community Survey 5-Year Estimates, Table B25014. e. Household digital access data were derived from 2021 American Community Survey 5-Year Estimates, Table S2801.
